# 40.2 MATERNAL IMMUNE ACTIVATION LEADS TO INCREASED LEVELS OF INFLAMMATORY CYTOKINES IN THE ABSENCE OF OVERT MICROGLIA ANOMALIES IN THE MIDBRAIN

**DOI:** 10.1093/schbul/sby014.165

**Published:** 2018-04-01

**Authors:** Ulrike Weber-Stadlbauer, Juliet Richetto, Marie Labouesse, Urs Meyer

**Affiliations:** 1 University of Zurich;; 2 College of Physicians and Surgeons, Columbia University/New York State Psychiatry

## Abstract

**Background:**

Inflammatory theories in schizophrenia have gained increasing recognition and acceptance in recent years. The evidence supporting a role of altered inflammatory processes in the etiology and pathophysiology of schizophrenia involves early-life exposure to infectious pathogens or inflammatory stimuli, increased expression of cytokines and other mediators of inflammation in the adult central nervous system (CNS) and periphery, as well as signs of glial anomalies. Given the role of dopaminergic deregulation in the pathophysiology of schizophrenia, inflammatory processes in the midbrain may contribute to dopamine abnormalities in the midbrain and its subcortical and cortical output regions. Here, we tested this hypothesis using an established neurodevelopmental mouse model with relevance to schizophrenia, namely the maternal immune activation (MIA) model.

**Methods:**

Pregnant C57BL6/N mice on gestation day 17 were treated with the viral mimetic polyriboinosinic-polyribocytidilic acid (poly(I:C)) or vehicle control solution. We then quantified the gene transcripts of an array of pro-inflammatory cytokines, acute phase proteins, and dopaminergic markers in the midbrain of MIA offspring (N=32) and control offspring (N= 32) at adult age. We also assessed the cell density of microglial cells expressing Iba1 and CD68 by immunohistochemistry to ascertain whether putative inflammatory changes are accompanied by microglia anomalies. Given the large sample sizes, we performed two-step recursive cluster analyses in order to identify possible subgroups of offspring that are characterized by “high” and “low” inflammatory profiles.

**Results:**

When considering the entire treatment group, MIA-exposed offspring displayed significantly increased expression of several inflammatory cytokines in the ventral midbrain, including IL-1b (p < 0.01), TNF-a (p < 0.01) and SERPINA3 (p < 0.01). These inflammatory changes occurred in the absence of overt microglia anomalies but were paralleled by changes in dopaminergic markers. The two-step cluster analyses further identified subgroups of MIA-exposed offspring that are characterized by a “high” (41 %, N = 13) and “low” (59 %, N = 19) inflammatory profiles. The “high” inflammatory subgroup of MIA-exposed offspring was defined by marked elevations of SERPINA3, IL-1β, IL-6, and TNFα mRNA levels (all p’s < 0.01).

**Discussion:**

Maternal immune activation during pregnancy causes persistent signs of inflammation in the offspring’s midbrain. In agreement with post-mortem studies in schizophrenia, these inflammatory abnormalities are clearly noticeable in a subgroup of MIA-exposed offspring only. Hence, prenatal immune activation may be one of the factors inducing lasting inflammatory changes relevant to (some cases of) schizophrenia and may contribute to dopaminergic dysfunctions in this disorder.

